# The Optimal Timing of Laparoscopic Cholecystectomy in Acute Cholecystitis: A Single-Center Study

**DOI:** 10.7759/cureus.38915

**Published:** 2023-05-11

**Authors:** Yasin Güneş, Emre Teke, Mehmet T Aydın

**Affiliations:** 1 General Surgery, Fatih Sultan Mehmet Training and Research Hospital, Istanbul, TUR; 2 General Surgery, Haydarpasa Numune Training and Research Hospital, Istanbul, TUR

**Keywords:** delayed laparoscopic cholecystectomy, prolonged early laparoscopic cholecystectomy, immediate laparoscopic cholecystectomy, early laparoscopic cholecystectomy, acute cholecystitis

## Abstract

Introduction

Early laparoscopic cholecystectomy (ELC) is a treatment option for acute cholecystitis (AC). However, the timing of ELC is controversial. Delayed laparoscopic cholecystectomy (DLC) continues to be a common practice. This study aims to determine the optimal timing of ELC in AC.

Materials and methods

Patients who underwent surgery for AC between 2014 and 2020 were divided into three groups: immediate laparoscopic cholecystectomy (ILC), prolonged ELC (pELC), and DLC. The demographic, laboratory, radiological findings, and postoperative results of all patients were retrospectively reviewed.

Results

The study included 178 patients, with 63 in the ILC group, 27 in the pELC group, and 88 in the DLC group. Postoperative outcomes, excluding hospital stay, were similar between the groups. The total hospital stay was significantly longer in the pELC and DLC groups (p<0.05). In addition, postoperative hospital stay was longer in the pELC group (p<0.05), and 17.7% of the patients who waited for delayed surgery experienced recurrent attacks during the interval period.

Conclusion

ILC is recommended in AC to minimize hospital stays.

## Introduction

Gallstone disease affects approximately 10% of the population [1], with 1%-4% of these patients developing complications related to gallstones, most commonly acute cholecystitis (AC) [2]. In the past, open early cholecystectomy was preferred over open delayed cholecystectomy due to the shortened hospital stay and recovery time without increased postoperative morbidity and mortality [3]. Early laparoscopic cholecystectomy (ELC) was contraindicated for patients with AC due to the high rates of morbidity, mortality, and bile duct injuries reported in early studies [4]. However, laparoscopic cholecystectomy (LC) has become the preferred treatment for patients with AC with the increase in experience in laparoscopic surgery, and recent meta-analyses have shown that ELC is superior [5].

The optimal timing for ELC is a topic of ongoing debate [6]. Several studies have defined ELC as surgery performed within 24 hours of admission [7-10], within 72 hours of admission [11-15], or within one week of admission [16]. Although the first 72-hour rule from symptom onset still applies, guidelines now recommend that surgery be performed as soon as possible, even if the duration of symptoms exceeds three days [17].

The objective of this study is to determine the most effective timing for ELC. Our findings will provide valuable insights into the optimal timing for ELC, which can help improve patient outcomes and reduce the burden of disease associated with AC.

## Materials and methods

Study population and design

This was a single-center retrospective study conducted at Istanbul Fatih Sultan Mehmet Training and Research Hospital, University of Health Sciences, between July 2014 and July 2020. Patients with AC who underwent LC were included in the study, whereas those who underwent percutaneous cholecystostomy (PC) were excluded. Patients with recurrent gallstone-related disease or multiple AC attacks were excluded from the study. AC diagnosis was made based on the Tokyo 2013/2018 guidelines [18,19]. Accordingly, patients with local inflammation findings (right upper quadrant pain/tenderness/palpable mass, and Murphy sign) and systemic inflammation findings (fever, C-reactive protein (CRP) elevation, and white blood cell (WBC) elevation) were further evaluated with upper abdominal ultrasonography, upper abdominal computed tomography or magnetic resonance cholangiopancreatography. Patients with characteristic AC findings (gallbladder wall thickness ≥ 4 mm, pericholecystic fluid, or sonographic Murphy positivity) on imaging were hospitalized with the diagnosis of AC.

Basic characteristics

Patients’ age, gender, clinical features, radiological findings, laboratory data, comorbidities, anesthesia risk, and Charlson Comorbidity Index (CCI) were recorded at the time of first admission. The presence of gallstones, gallbladder wall thickness, gallbladder diameter, pericholecystic fluid, and AC grade according to the Tokyo 2018 guidelines [19] were recorded. The CCI of each patient was calculated by giving points from 1 to 6 for age and comorbid diseases. Risk classification by the American Society of Anesthesiologists (ASA) was used to evaluate the anesthesia risk. Wall thickness ≥ 4 mm was considered as an increase in wall thickness, and transverse diameter ≥ 4 cm was considered as a hydropic gallbladder. The laboratory data recorded from the admission charts included WBC count and CRP levels in the first admission. AC was classified as grade 1 (mild), grade 2 (moderate), and grade 3 (severe) using the Tokyo 2018 guidelines [19] by evaluating the preoperative findings of the patients.

Timing of surgery and surgical procedure

We divided patients into three groups according to the operation time. Patients who underwent cholecystectomy in the first 24 hours of hospitalization constituted the immediate laparoscopic cholecystectomy (ILC) group, patients who underwent cholecystectomy from 24 hours to eight days constituted the prolonged early laparoscopic cholecystectomy (pELC) group, and patients who received broad-spectrum intravenous antibiotic therapy at hospitalization and were operated on at the earliest four weeks after discharge constituted the delayed laparoscopic cholecystectomy (DLC) group.

Surgical procedures were classified as LC, conversion to open cholecystectomy (CC). LC was performed using the standard four-trocar technique. Conversion cholecystectomy was performed through a right subcostal incision.

Operative outcomes

Primary outcomes included the type and duration of surgery, as well as any complications during surgery. Secondary outcomes included postoperative complications, mortality rates, total and postoperative hospital stays, and Clavien-Dindo classification for complications.

Statistical analysis

The Statistical Package for the Social Sciences (SPSS) version 22 (IBM SPSS Statistics, Armonk, NY, USA) was used for statistical analysis and evaluation of the findings obtained in the study. Descriptive statistical methods (mean, standard deviation (SD), frequency, and percentage) were used while evaluating the study data. The suitability of quantitative parameters to the normal distribution was evaluated with the Shapiro-Wilk test. Firstly, comparisons were made between the three groups. Subsequently, statistically significant results were compared between the two groups. A one-way ANOVA test was used for comparing normally distributed parameters between the groups. The Kruskal-Wallis test and Mann-Whitney U test were used for comparisons of non-normally distributed parameters between the groups. The Pearson chi-square test, Fisher’s exact test, and Fisher-Freeman-Halton test were used for the comparison of qualitative data. Significance was evaluated at the p<0.05 level.

## Results

**Figure 1:**
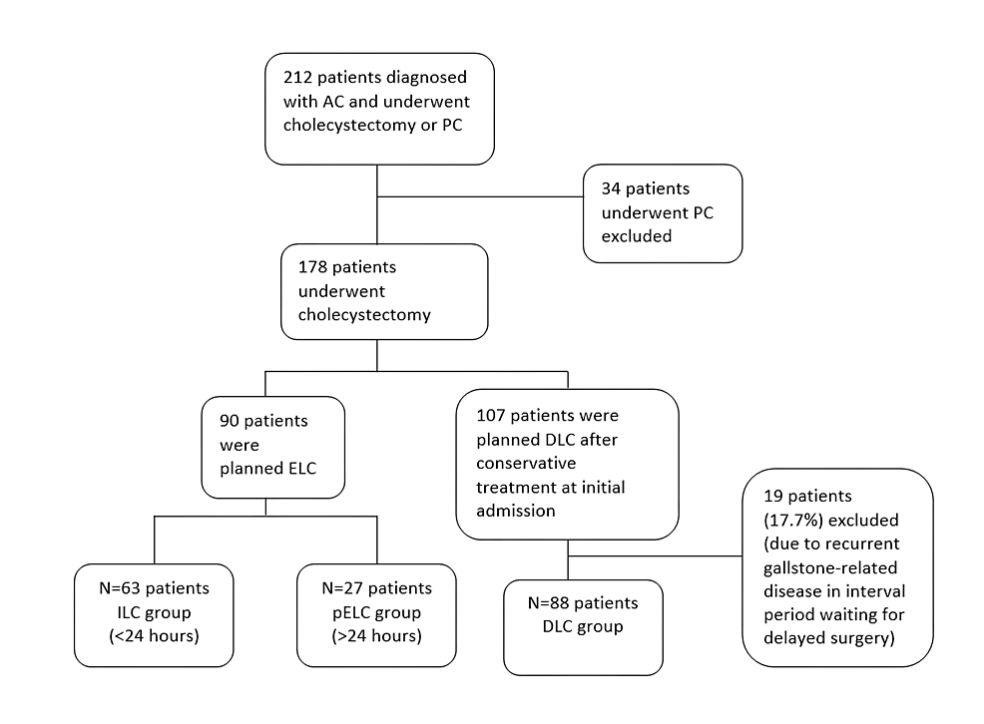
Flow Diagram of Patients Enrolled AC: acute cholecystitis, PC: percutaneous cholecystostomy, ILC: immediate laparoscopic cholecystectomy, pELC: prolonged early laparoscopic cholecystectomy, DLC: delayed laparoscopic cholecystectomy

The number of patients diagnosed with AC and treated with LC or PC was 212. Thirty-four patients were treated with PC. A total of 178 patients underwent cholecystectomy. Ninety patients performed ELC. Patients who underwent ELC were divided into two groups. The ILC group consisted of 63 patients, and the pELC group consisted of 27 patients. Overall, 107 patients were planned for DLC after conservative treatment at initial admission. However, 19 patients were excluded due to recurrent gallstone-related disease in an interval period waiting for delayed surgery. The DLC group consisted of 88 patients (Figure 1). The mean time from hospitalization to surgery in the pELC group was 3.5 days. In the DLC group, the mean time until the surgery was 88.6 days.

Of the patients in the ILC group, pELC group, and DLC group, 58.7%, 37%, and 54.5%, respectively, were female (p=0.166). The mean age was 47.1±14.4 years in the ILC group, 48.6±11.93 years in the pELC group, and 57.8±13.11 years in the DLC group (p<0.001). There was a statistically significant difference between the ILC and DLC groups and also the pELC and DLC groups in subgroup analyses (p<0.05). The mean CCI was 0.9±1.37 in the ILC group, 1.2±1.28 in the pELC group, and 1.3±1.31 in the DLC group (p=0.058). Of the patients in the DLC group, 21.6% were ASA 3, and of the patients in the pELC group, 11.1% were ASA 4 (p=0.042). There was a statistically significant difference only between the pELC and DLC groups in subgroup analyses (p<0.05). Also, patients with grade 2 AC were significantly higher in the DLC group (p<0.001). There was a statistically significant difference between the ILC and DLC groups and also the pELC and DLC groups in subgroup analyses (p<0.05). The mean symptom duration was 2.2±2.6 days in the ILC group, 2.08±2.27 days in the pELC group, and 2.9±2.76 days in the DLC group (p=0.085). Patients with symptom duration of >3 days were 14.3% in the ILC group, 22.2% in the pELC group, and 31.8% in the DLC group (p=0.112). There was a statistically significant difference only between the ILC and DLC groups in subgroup analyses (p<0.05) (Table 1).

**Table 1:** Baseline and Clinical Characteristics Comparing ILC, pELC, and DLC Groups ^1^One-way ANOVA test, ^2^Kruskal-Wallis test, ^3^Fisher-Freeman-Halton test, ^a^ILC and DLC, ^b^pELC and DLC, *significant at p<0.05 ILC: immediate laparoscopic cholecystectomy, pELC: prolonged early laparoscopic cholecystectomy, DLC: delayed laparoscopic cholecystectomy, CCI: Charlson Comorbidity Index, WBC: white blood cell, CRP: C-reactive protein, ASA: American Society of Anesthesiologists, SD: standard deviation

		ILC (n=63)	pELC (n=27)	DLC(n=88)	P-value
		Mean±SD	Mean±SD	Mean±SD	
Age (years)		47.1±14.4	48.6±11.93	57.8±13.11	^1^<0.001*^a,b^
CCI		0.9±1.37	1.2±1.28	1.3±1.31	^2^0.058
Symptom duration (days)		2.2±2.6	2.08±2.27	2.9±2.76	^2^0.085
Gallbladder wall thickness (mm)		4.5±1.72	4.7±1.78	5.2±1.56	²0.004*^a^
Gallbladder diameter (cm)		3.4±0.82	3.8±0.95	3.8±1.04	²0.045*^a^
WBC (×10^9^/L)		12890±4795	13511±3814	13977±4771	²0.345
CRP (mg/dL)		7.3±8.04	7.6±8.19	10.2±8.8	²0.135
		Number (%)	Number (%)	Number (%)	
Gender	Male	26 (41.3)	17 (63)	40 (45.5)	^3^0.166
	Female	37 (58.7)	10 (37)	48 (54.5)	
Symptom duration	≤3 days	54 (85.7)	21 (77.8)	60 (68.1)	^3^0.043*^a^
	>3 days	9 (14.3)	6 (22.2)	28 (31.8)	
Gallbladder wall thickness	<4 mm	16 (25.4)	6 (22.2)	11 (12.5)	^3^0.105
	≥4 mm	47 (74.6)	21 (77.8)	77 (87.5)	
Gallbladder diameter	<4 cm	45 (71.4)	16 (59.2)	43 (48.9)	^3^0.037*^a^
	≥4 cm	18 (28.6)	11 (40.8)	45 (51.1)	
Pericholecystic fluid	No	39 (61.9)	17 (63)	46 (50.4)	^3^0.411
	Yes	24 (38.1)	10 (37)	42 (37.6)	
ASA	1	28 (44.4)	10 (37)	28 (31.8)	^3^0.042*^b^
	2	25 (39.7)	12 (44.4)	41 (46.6)	
	3	8 (12.7)	2 (7.4)	19 (21.6)	
	4	2 (3.2)	3 (11.1)	-	
Grade	1 (mild)	44 (69.8)	15 (65.2)	36 (40.9)	^3^<0.001*^a,b^
	2 (moderate)	17 (27)	7 (30.4)	52 (59.1)	
	3 (severe)	2 (3.2)	1 (4.3)	-	

Gallbladder wall thickness was 4.5±1.72 mm in the ILC group, 4.7±1.78 mm in the pELC group, and 5.2±1.56 mm in the DLC group (p=0.004). There was a statistically significant difference only between the ILC and DLC groups in subgroup analyses (p<0.05). Of the patients in the ILC group, pELC group, and DLC group, 74.6%, 77.8%, and 87.5%, respectively, have gallbladder wall thickness of ≥4 mm (p=0.105). Gallbladder diameter was 3.4±0.82 cm in the ILC group, 3.8±0.95 cm in the pELC group, and 3.8±1.04 cm in the DLC group (p=0.045). There was a statistically significant difference only between the ILC and DLC groups in subgroup analyses (p<0.05). Of the patients in the ILC group, pELC group, and DLC group, 28.6%, 40.8%, and 51.1%, respectively, have a gallbladder diameter of ≥4 cm (p=0.037). There was a statistically significant difference only between the ILC and DLC groups in subgroup analyses (p<0.05) (Table 1).

WBC was 12890±4795×10^9^/L in the ILC group, 13511±3814×10^9^/L in the pELC group, and 13977±4771×10^9^/L in the DLC group (p=0.345). CRP was 7.3±8.04 mg/dL in the ILC group, 7.6±8.19 mg/dL in the pELC group, and 10.2±8.8 mg/dL in the DLC group (p=0.135) (Table 1).

As potential factors in planning delayed cholecystectomy, higher age, higher gallbladder wall thickness, higher gallbladder diameter, gallbladder diameter of ≥4 cm, symptom duration of >3 days, grade 2 AC, and ASA score of ≥3 were statistically significant. In addition, higher CCI and higher symptom duration were marginally significant.

It was seen that there was no difference in conversion rate, operation time, and complication between the groups. There were no perioperative complications such as bile duct injury among the three groups. There were five postoperative complications in the ILC group; three patients had obstructive jaundice, one patient had acute biliary pancreatitis, and one patient had gastrointestinal bleeding. There were two postoperative complications in the pELC group; one patient had obstructive jaundice, and another patient had acute biliary pancreatitis. There were five postoperative complications in the DLC group; three patients had obstructive jaundice, and two patients had acute biliary pancreatitis (p=0.780). Three patients in the ILC group and five patients in the DLC group were converted to open cholecystectomy. There was no patient in the pELC group converted to open cholecystectomy (p=0.628). The mean operation time was 75.8±31.57 minutes in the ILC group, 82±27.7 minutes in the pELC group, and 79.7±33.73 minutes in the DLC group (p=0.382) (Table 2).

**Table 2:** Intraoperative and Postoperative Results Comparing ILC, pELC, and DLC Groups ^1^Kruskal-Wallis test, ^2^Fisher-Freeman-Halton test, ^a^ILC and pELC, ^b^ILC and DLC, ^c^pELC and DLC, *significant at p<0.05 ILC: immediate laparoscopic cholecystectomy, pELC: prolonged early laparoscopic cholecystectomy, DLC: delayed laparoscopic cholecystectomy, LC: laparoscopic cholecystectomy, CC: conversion to open cholecystectomy, SD: standard deviation

		ILC (n=63)	pELC (n=27)	DLC(n=88)	P-value
		Mean±SD	Mean±SD	Mean±SD	
Overall hospital stay (days)		2.7±1.5	7.2±3.43	6.2±3.84	^1^<0.001*^a,b^
Preoperative hospital stay (days)		0.6±0.48	3.5±1.5	3.7±1.68	^1^<0.001*^a,b^
Postoperative hospital stay (days)		2.1±1.38	3.6±3.47	2.4±2.94	^1^0.02*^a,c^
Operation time (minute)		75.8±31.57	82±27.7	79.7±33.73	^1^0.382
		Number (%)	Number (%)	Number (%)	
Surgery type	LC	60 (95.2)	27 (100)	83 (94.3)	^2^0.628
	CC	3 (4.8)	-	5 (5.7)	
Complication	Intraoperative	-	-	-	^2^0.780
	Postoperative	5 (7.9)	2 (7.4)	5 (5.6)	
Clavien-Dindo classification	1	-	-	-	^2^0.838
	2	4 (6.3)	1 (3.7)	4 (4.5)	
	3a	-	-	-	
	3b	1 (1.6)	1 (3.7)	1 (1.1)	
	4a	-	-	-	
	4b	-	-	-	
	5	-	-	-	
Mortality		-	1	-	^2^0.506

The total hospital stay was 2.7±1.5 days in the ILC group, 7.2±3.43 days in the pELC group, and 6.2±3.84 days in the DLC group (p<0.001). Preoperative hospital stay was 0.6±0.48 days in the ILC group, 3.5±1.5 days in the pELC group, and 3.7±1.68 days in the DLC group (p<0.001). Postoperative hospital stay was 2.1±1.38 days in the ILC group, 3.6±3.47 days in the pELC group, and 2.4±2.94 days in the DLC group (p=0.02). The total hospital stay was statistically significantly higher in the pELC and DLC groups in subgroup analyses (p<0.05). Preoperative hospital stay was higher in the pELC and DLC groups in subgroup analyses (p<0.05). Postoperative hospital stay was higher in the pELC group in subgroup analyses (p<0.05). There was one mortality due to cardiac and respiratory disorders in the pELC group (p=0.506) (Table 2).

## Discussion

LC is accepted as the gold standard for AC [3]. There are two preferred treatment options: surgeons may opt for early cholecystectomy in the first attack or delayed cholecystectomy after conservative treatment [4]. However, there is no clear consensus in the literature regarding the definition of early treatment. The definition of early LC varies widely, ranging from LC performed within 24 hours to LC performed up to 10 days after diagnosis [20]. In our study, ELC was performed up to eight days after admission. According to Strasberg [5], early surgery is defined as surgery performed anywhere from 24 hours to seven days after the onset of symptoms or the time of diagnosis. The guideline published by the European Association for Endoscopic Surgery in 2012 defines early treatment as ranging from four to seven days after the onset of symptoms [6]. The World Society of Emergency Surgery guidelines recommend ELC within 10 days from the onset of symptoms, with earlier surgery resulting in shorter complications and hospital stays [21]. Delayed treatment is defined as surgery performed 6-12 weeks after conservative treatment with antibiotics, analgesics, and fluid replacement [6,17].

In the current study, no significant differences were observed between the groups in postoperative outcomes, except for hospital stay. Surgery after conservative treatment did not provide any advantage. Many meta-analyses in the literature have compared the outcomes of ELC and DLC. A meta-analysis of six randomized clinical trials comparing ELC and DLC for patients with AC found no difference in bile duct injury and other serious adverse effects, CC, and mortality between the groups. However, the total hospital stay was longer in patients undergoing DLC [22]. Another study found no difference in complication rates and CC between the groups, but the operation time was shorter and the total hospital stay was longer in the delayed cholecystectomy group [23]. A meta-analysis by Cao et al. [1] including 77 studies found that mortality, complications, CC, and total hospital stay were significantly lower in patients undergoing ELC. Lyu et al. [24] reported no difference in bile duct injury, bile leakage, wound infection, and CC between the groups but found that the operation time was longer in the ELC group and the total hospital stay was longer in the DLC group.

In the guidelines of the European Association for Endoscopic Surgery, 17.5% of the patients who planned for delayed surgery required emergency surgery due to recurrent symptoms during the interval period. In this patient subgroup, the conversion rate to open surgery was 45% [6]. In the meta-analysis published by Papi et al. [25], the percentage of patients who did not respond to conservative treatment was 9.3%, and the percentage of those experiencing recurrent symptoms in the interval period was 14.9%. The recurrence rate in the interval period was reported as 18.3% in the study by Gurusamy et al. [22]. In the guideline published by the World Society of Emergency Surgery in 2016, 14% in six weeks, 19% in 12 weeks, and 29% in one year reported recurrent symptoms in patients who were discharged home after conservative treatment for AC [21]. In our study, the recurrence rate was 17.7% in four weeks, similar to the literature.

As many studies and meta-analyses have shown, delayed surgery does not provide any benefits. Generally, there is a consensus that surgery should be performed during the first admission. The optimum timing of ELC is currently the most controversial issue in LC [20]. The large database study of Zafar et al. [20] found that ELC performed within the first 48 hours reduced morbidity, mortality, and hospital stay. Similarly, the study by Banz et al. [26] using a large database found that delaying ELC beyond 48 hours after admission resulted in increasing conversion to open surgery, more complications, and longer postoperative hospital stays. Brooks et al. [27] found an increase in morbidity in ELC performed after day 4. Similar to our study, Stevens et al. [28] found that ELC performed after 24 hours prolonged the hospital stay, and there was no difference in other outcomes. A major criticism of these studies is that patients who had surgery later may have been sicker than baseline and therefore had worse outcomes [20]. In another recent study, the total hospital stay and postoperative hospital stay were longer in the prolonged laparoscopic cholecystectomy group (≥72 hours), similar to our study [29]. In a different study, cholecystectomy within three days of admission reduced CC, biliary complications, and hospital stay [30].

Our study has some limitations that need to be acknowledged. Firstly, it is important to note that our study is retrospective in nature. As is commonly known, retrospective studies are susceptible to potential issues with group standardization. In our study, we observed differences in preoperative characteristics between the groups, which may have influenced the results. Secondly, the sample size of our study is relatively small. Thus, larger prospective studies are needed to provide a more comprehensive evaluation of the subject matter.

## Conclusions

Our study found that the total hospital stay was the shortest in the ILC group. The ILC and DLC groups had the shortest postoperative hospital stay. The postoperative hospital stay was the longest in the pELC group. The pELC and DLC groups had the longest total hospital stay. Therefore, we do not recommend pELC. We also do not recommend DLC due to the prolonged total hospital stay and the occurrence of recurrent gallstone-related disease in 17.7% of patients during the interval period. We recommend ILC for the treatment of AC to minimize hospital stays based on these findings.
